# The Effect of Gold Nanoparticles in Sodium Alginate on the Biochemical Characteristics of Garden Cress

**DOI:** 10.3390/molecules31081373

**Published:** 2026-04-21

**Authors:** Miłosz Rutkowski, Damian Duda, Ewa Godos, Wojciech Makowski, Emilia Bernaś, Karen Khachatryan, Andrzej Kalisz, Agnieszka Sękara, Gohar Khachatryan

**Affiliations:** 1 Centre for Innovation and Research on Prohealthy and Safe Food, University of Agriculture in Krakow, Balicka 104, 31-120 Krakow, Poland; 2 Faculty of Food Technology, University of Agriculture in Krakow, Balicka 122, 31-120 Krakow, Poland; damian.duda@student.urk.edu.pl (D.D.); emilia.bernas@urk.edu.pl (E.B.); karen.khachatryan@urk.edu.pl (K.K.); 3 Faculty of Biotechnology and Horticulture, University of Agriculture in Krakow, 29 Listopada 54, 31-425 Krakow, Poland; ewa.godos@urk.edu.pl (E.G.); wojciech.makowski@urk.edu.pl (W.M.); andrzej.kalisz@urk.edu.pl (A.K.); agnieszka.sekara@urk.edu.pl (A.S.)

**Keywords:** nanotechnology, green chemistry, bioactive compounds, biopolymers, horticulture, vegetables, agriculture

## Abstract

Gold nanoparticles (AuNPs) have numerous applications in science and industry. Therefore, their potential phytotoxicity should be investigated. Garden cress (*Lepidium sativum* L.) is a useful model plant for assessing the effects of chemicals released into the environment. The aim of this study was to prepare alginate gels containing AuNPs for plant exposure experiments, evaluate their physicochemical properties, and determine their effects on selected biochemical parameters of garden cress seedlings. Gold nanoparticles were synthesized in sodium alginate at an initial concentration of 50 mg/L, using xylose and maltose as reducing agents. The gels were diluted with distilled water to obtain AuNP concentrations of 5 and 25 mg/L. Garden cress seeds were placed on filter paper soaked with the tested formulations, while distilled water and sodium alginate solutions without AuNPs served as controls. After 5 days of incubation at 20 °C under light conditions, the plant material was collected and selected bioactive compounds were determined. AuNP-containing gels significantly affected the biochemical status of the seedlings. In particular, AuNPs synthesized with xylose at 25 mg/L significantly increased the contents of photosynthetic pigments and total polyphenolic compounds. All tested AuNP formulations increased the antioxidant activity of seedlings, suggesting the activation of abiotic stress-related defense responses, however, direct markers of oxidative damage were not assessed in the present study. Overall, the results indicate that alginate-based AuNPs can modify selected biochemical parameters in garden cress seedlings, and these effects depend on nanoparticle concentration and reducing sugar used during synthesis, which may be relevant for the future development of plant-targeted nanomaterials for agricultural applications.

## 1. Introduction

Nanotechnology is an interdisciplinary field concerned with the design and application of materials and systems at the nanoscale, typically within the range of 1–100 nm. Owing to the unique size-dependent properties of nanomaterials, nanotechnology has found applications in diverse sectors, including agriculture, food processing, medicine, energy, and oil- and gas-related technologies [1,2,3,4,5,6,7,8]. Nanoparticles exhibit specific electromagnetic, antimicrobial, or optical properties that result from their size, specific area, agglomeration or shape [9,10,11,12]. Gold nanoparticles (AuNPs) are characterized by a large surface-to-volume ratio and high stability. Their optical properties allow them to convert energy into heat. Gold nanoparticles can bind to amino acids or peptides. Due to their ease of binding to drugs, they are also being explored as potential carriers in biomedical research. AuNPs are used in detection, including diagnostic analyses using fluorometric, colorimetric, and spectroscopic methods [13,14,15,16]. In agricultural sciences, gold nanoparticles are the subject of contemporary diagnostic studies on the effects of nanostructures on plants. It has been demonstrated that nanoparticles can modify the morphological characteristics of plant cell structures such as chloroplasts [17]. Chemical synthesis based on the principles of green chemistry is an effective method for obtaining AuNPs. Modifying the synthesis reaction conditions, such as temperature, the type of solvents and sugars used, and pH, directly influences the physicochemical properties (shape and size) and biological activity of gold nanoparticles. Natural biopolymers can act as carriers and stabilizers for the resulting AuNPs [18]. Sodium alginate is a polysaccharide that can be enriched with nanoparticles. Alginate consists of β-D-mannuronic acid and α-L-guluronic acid residues. The chemical structure of sodium alginate is linked by glycosidic bonds. It is biodegradable. In the food industry, it is used as an ingredient in the development of innovative packaging coatings [19,20]. Composites containing metal nanoparticles may in the future be used as raw materials for the production of innovative horticultural preparations. In green nanosystems, reducing sugars may play a dual role as reductants and surface-active stabilizing agents; therefore, in plant-response studies, the biological effects of the final formulation may reflect not only the nanoparticle core itself, but also the matrix- and surface-associated components derived from the synthesis route [18,21]. In modern agrochemical research, alginate composites with nanoparticles are used to assess phytotoxicity to living organisms, such as vegetable plants [22]. Garden cress (*Lepidium sativum* L.) is a nutritionally valuable vegetable, characterized by notable antioxidant activity, which makes it a useful model for studies on stress-related biochemical responses [23,24]. Botanically, garden cress belongs to the Brassicaceae family. It is a valuable source of numerous nutrients, such as dietary fiber, proteins, carbohydrates, phenols like flavonoids, and vitamins [24]. In agrochemical studies, garden cress is used both as a target plant and a test organism. Research conducted by Jośko and Oleszczuk [25] indicated a variety of experimental methods for treating garden cress with nanoparticles, while Mošenoka et al. [26] used garden cress as a model in analyses of the genotoxicity of nanoparticles in hydroponic conditions [26]. Studies on the phytotoxicity of silver nanoparticles were also conducted using garden cress seeds [27]. The widespread use of garden cress as a research object indicates its validity in relation to the conducted experiment. Recent studies have also shown that the effects of AuNPs on plants depend on plant species, exposure route, concentration, and duration of treatment, and may include both stress-related and potentially stimulatory responses [28,29,30,31]. At the same time, the limited number of reports assessing the phytotoxicity of AuNPs to plants determines the need to expand the information in the scientific literature.

The aim of the study was to obtain alginate gels containing AuNPs as formulations for investigating the effect of gold nanoparticles, evaluate their physicochemical properties, and assess their effect on selected biochemical parameters in garden cress (*Lepidium sativum* L.) seedlings. The experimental study was designed to test the following hypotheses: (I) the use of maltose or xylose as non-toxic reducing agents allows the production of gold nanoparticles in a sodium alginate matrix; (II) exposure of garden cress seedlings to alginate-based AuNPs alters stress-related biochemical parameters including antioxidant activity, photosynthetic pigments, and polyphenolic compounds; (III) the plant response depends on both the concentration of AuNPs and the type of reducing sugar used during nanoparticle synthesis.

## 2. Results

### 2.1. Physicochemical Properties of Alginate Gels with Gold Nanoparticles

#### 2.1.1. Transmission Electron Microscopy (TEM), Particle Size Distribution, and EDS

**Figure 1:**
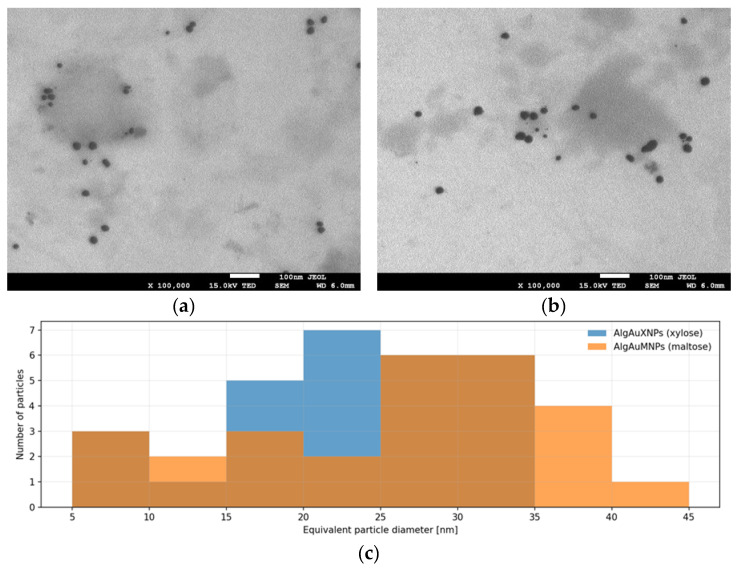
TEM micrographs and particle size distribution of alginate-based gold nanoparticles synthesized using different reducing sugars. The upper panels show representative micrographs of AlgAuXNPs ((**a**), xylose) and AlgAuMNPs ((**b**), maltose). The lower panel (**c**) presents the corresponding particle size distributions; the blue histogram represents AlgAuXNPs (xylose), while the orange histogram represents AlgAuMNPs (maltose). AlgAuXNPs showed a narrower size distribution and lower polydispersity, whereas AlgAuMNPs exhibited slightly larger and more polydisperse particles. The scale bar corresponds to 100 nm.

Transmission electron microscopy (TEM) confirmed the successful formation of gold nanoparticles in both alginate-based systems synthesized using different reducing sugars. In both samples, the nanoparticles were predominantly quasi-spherical and well-embedded within the alginate matrix; however, clear differences in particle size distribution and dispersity were observed depending on the reducing agent used. The AlgAuXNPs sample, synthesized using xylose, exhibited a more homogeneous nanoparticle population, with most particles falling within the 20–30 nm range and a mean equivalent diameter of 23.1 ± 7.2 nm. In contrast, the AlgAuMNP sample, obtained using maltose, displayed slightly larger particles overall, with a mean equivalent diameter of 25.8 ± 10.4 nm, together with a visibly broader size distribution. This difference was also reflected in the higher coefficient of variation for AlgAuMNPs (40.2%) compared with AlgAuXNPs (31.2%), as well as in the greater proportion of particles exceeding 30 nm (40.7% vs. 21.4%). Representative TEM micrographs and the corresponding particle size distributions are shown in Figure 1, while the numerical summary of the semi-automated particle analysis is presented in Table 1.

The microscopic observations were consistent with the UV–Vis data, in which the AlgAuMNP sample showed a red-shifted surface plasmon resonance band compared with AlgAuXNPs. This finding indicates that the type of reducing sugar influenced not only the reduction process itself, but also the final morphology and dispersity of the resulting nanostructures, which is in line with previous observations reported for starch-based nanocomposites [32].

**Figure 2:**
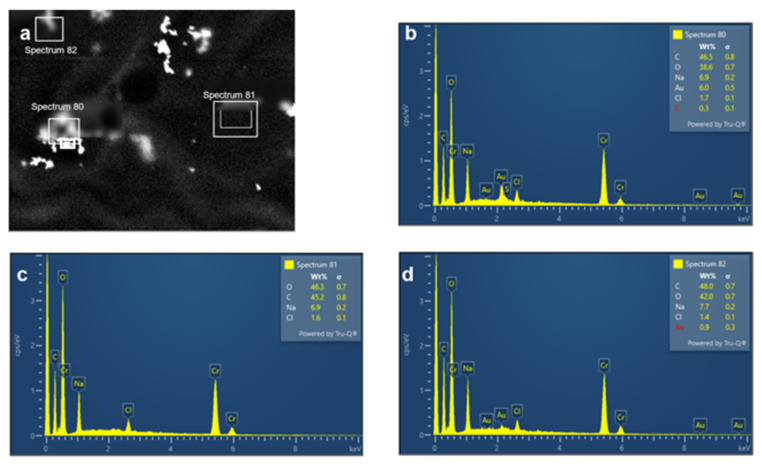
Representative TEM-EDS analysis of the AlgAuXNP sample. The upper-left panel (**a**) shows the TEM micrograph with the three selected EDS acquisition regions. The remaining three panels show the corresponding spectra acquired from an Au-rich region ((**b**), spectrum 80), a background region ((**c**), spectrum 81), and a low-Au-signal region ((**d**), spectrum 82). The EDS results confirm the presence of Au in the analyzed sample.

Representative energy-dispersive X-ray spectroscopy (EDS) analysis further confirmed the presence of gold in the analyzed material. In the nanoparticle-rich region, the recorded spectrum showed clear Au signals, whereas the background region exhibited no detectable Au contribution and the third selected region showed only a weak Au signal. These results support the interpretation that the bright electron-dense domains observed in the TEM images correspond to Au-containing nanostructures embedded within the alginate-based matrix (Figure 2) [33].

#### 2.1.2. Dynamic Light Scattering (DLS) and Zeta Potential

Dynamic light scattering analysis showed that the hydrodynamic diameters of the nanoparticles were markedly greater than the dimensions observed by TEM. For the AlgAuXNP sample, the hydrodynamic diameter was 58 nm, whereas for AlgAuMNPs, it was 82 nm. When compared with the median TEM diameters obtained from the particle size-distribution analysis (23.9 nm for AlgAuXNPs and 28.3 nm for AlgAuMNPs), these values indicate that the apparent particle size in the hydrated state was more than twice that of the metallic core.

The zeta potential values were negative for both formulations, amounting to −38.5 mV for AlgAuXNPs and −32.5 mV for AlgAuMNPs. The more negative zeta potential observed for the xylose-reduced sample indicates greater electrostatic stabilization of the colloidal system. In turn, the combination of a smaller hydrodynamic diameter and a more negative zeta potential suggests that AlgAuXNPs formed a more stable and better-dispersed nanoparticle system than AlgAuMNPs.

#### 2.1.3. Ultraviolet–Visible (UV–Vis) Spectroscopy

**Figure 3:**
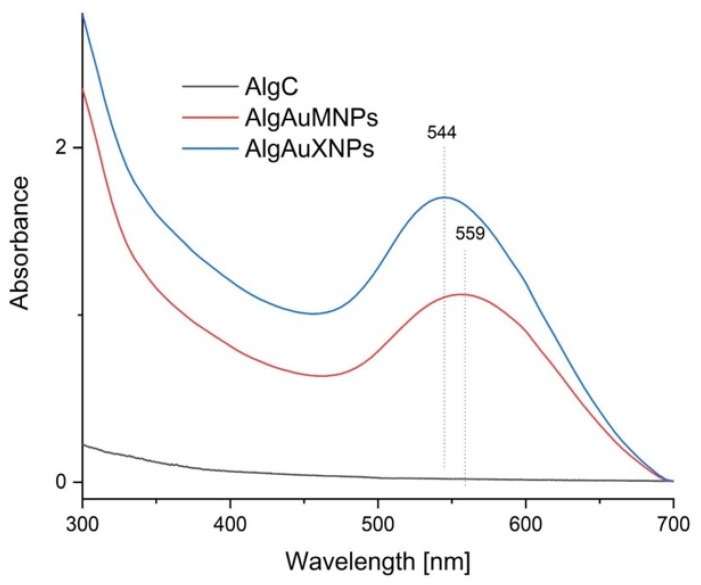
UV–Vis absorption spectra of the analyzed alginate gels: AlgC (control sodium alginate gel without nanoparticles), AlgAuMNPs (nanocomposite with maltose-reduced AuNPs, λmax = 559 nm), and AlgAuXNPs (nanocomposite with xylose-reduced AuNPs, λmax = 544 nm).

Figure 3 presents the UV–Vis absorption spectra of the alginate gels. The control sample (AlgC) did not exhibit any absorption peak in the visible region. In contrast, the nanocomposites showed distinct surface plasmon resonance (SPR) bands characteristic of gold nanoparticles. The spectrum for AlgAuXNPs (synthesized with xylose) displayed an absorption maximum centered at 544 nm, whereas the AlgAuMNP sample (synthesized with maltose) exhibited a red-shifted peak at 559 nm [34,35]. The position and width of the SPR band are closely related to the particle size and polydispersity; generally, a shift toward longer wavelengths indicates the formation of larger particles or aggregates. The observed red shift for AlgAuMNPs suggests that using maltose as a reducing agent results in larger gold nanoparticles compared to xylose. This difference can be attributed to the different reducing powers of the sugars; xylose, being a monosaccharide and a stronger reducing agent, likely facilitates a faster nucleation rate, leading to smaller particles. These results align with findings by Janik et al. [18], who reported that the choice of conditions and reducing agents significantly affects the geometric parameters of metallic nanoparticles in polysaccharide matrices. The broadness of the peaks, particularly for AlgAuXNPs, indicates a polydisperse distribution of particle sizes [18,32,36].

#### 2.1.4. Attenuated Total Reflection-Fourier Transform Infrared Spectroscopy (ATR-FTIR)

**Figure 4:**
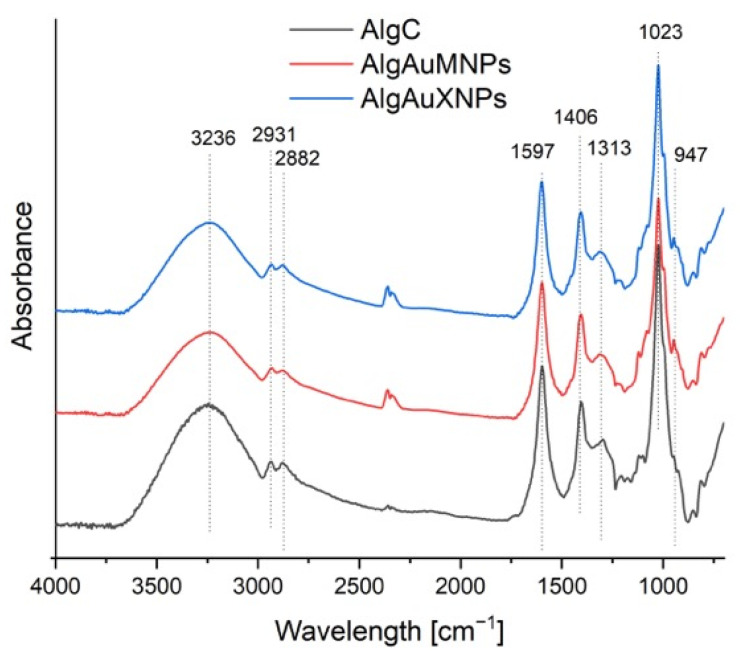
ATR-FTIR spectra of the analyzed alginate gels.

The FTIR-ATR spectra (Figure 4) of the two alginate-based nanocomposites containing gold nanoparticles, synthesized using xylose (AlgAuXNPs) and maltose (AlgAuMNPs) as reducing agents, confirmed the successful formation of the composites while revealing subtle differences attributable to the nature of the sugar reductant. The spectra exhibited all characteristic bands of the alginate matrix. A broad band centered at approximately 3236 cm^−1^ was attributed to O–H stretching vibrations from the alginate backbone, residual water, and hydroxyl groups of the incorporated sugars [37,38]. The weaker bands near 2931 and 2882 cm^−1^ corresponded to aliphatic C–H stretching. The key functional groups of alginate were evidenced by the asymmetric and symmetric stretching vibrations of the ionized carboxylate groups (–COO^−^) at approximately 1597 cm^−1^ and 1406 cm^−1^, respectively [38,39]. The strong and complex band around 1023 cm^−1^ was assigned to C–O–C glycosidic linkages, the intensity of which is influenced by the presence of the sugar-derived moieties [40]. The primary spectral differences between AlgAuXNPs and AlgAuMNPs, though minor, were observable in the regions associated with hydroxyl and carboxylate interactions. The shape and breadth of the O–H stretching envelope (~3236 cm^−1^) varied slightly between the two samples, suggesting differences in the hydrogen-bonding network within the nanocomposite. The findings align with the literature on green synthesis employing sugar reductants. As noted by Dwivedi & Gopal [41], sugars not only facilitate the reduction of metal ions but also stabilize the resulting nanoparticles through surface adsorption, leaving residual hydroxyl groups that can enhance compatibility with polysaccharide matrices like alginate. This is often reflected in FTIR-ATR spectra by reinforced absorption in the 1000–1100 cm^−1^ region, associated with C–O stretching and modifications in the carbonyl region. The FTIR-ATR data (Figure 4) confirmed that both maltose and xylose effectively produced stabilized AuNPs within the alginate without degrading the polymer’s primary structure. Minor spectral discrepancies indicate that selecting between aldose reductants like maltose and xylose allows for precise tuning of the nanocomposite’s interfacial chemistry, and consequently, its colloidal stability—a critical parameter for demanding biomedical applications [42,43].

### 2.2. Morphological Characteristics of Seedlings

**Figure 5:**
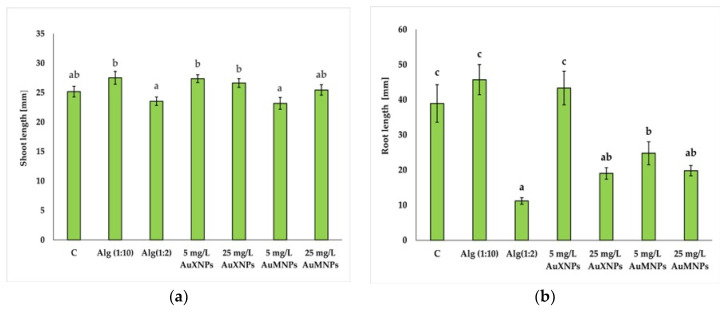
Average shoot (**a**) and root (**b**) lengths of garden cress. Treatments: C—negative control—distilled water, Alg(1:10)—positive control—sodium alginate without gold nanoparticles, diluted 10-fold, Alg(1:2)—positive control—sodium alginate without gold nanoparticles, diluted 2-fold, 5 mg/L AuXNPs—solution with gold nanoparticles obtained by using xylose as a reducing agent at a concentration of 5 mg/L, 25 mg/L AuXNPs—solution with gold nanoparticles obtained by using xylose as a reducing agent at a concentration of 25 mg/L, 5 mg/L AuMNPs—solution with gold nanoparticles obtained by using maltose as a reducing agent at a concentration of 5 mg/L, 25 mg/L AuMNPs—solution with gold nanoparticles obtained by using maltose as a reducing agent at a concentration of 25 mg/L. Statistically, differences marked with letters (a, b, c) differ significantly at *p* ≤ 0.05 according to Fisher’s test. Standard error was used to describe the obtained results.

No significant differences in shoot length were observed between the individual treatments and the negative control (C) in garden cress seedlings (Figure 5a). However, the mean shoot length was significantly greater in seedlings treated with both concentrations of AuNPs synthesized using xylose (5 and 25 mg/L AuXNPs), as well as in the lower alginate control (Alg(1:10)), compared with seedlings treated with 5 mg/L AuMNPs and the higher alginate control (Alg(1:2)).

In contrast, root length was more sensitive to the applied treatments (Figure 5b). A significant reduction in mean root length, relative to the negative control, was observed after treatment with both concentrations of AuNPs synthesized using maltose (5 and 25 mg/L AuMNPs), AuNPs synthesized using xylose at 25 mg/L, and the higher alginate control (Alg(1:2)). Only the treatment with 5 mg/L AuXNPs and the lower alginate control (Alg(1:10)) did not significantly affect the root length compared with the negative control.

### 2.3. Biochemical Characteristics of Garden Cress

**Figure 6:**
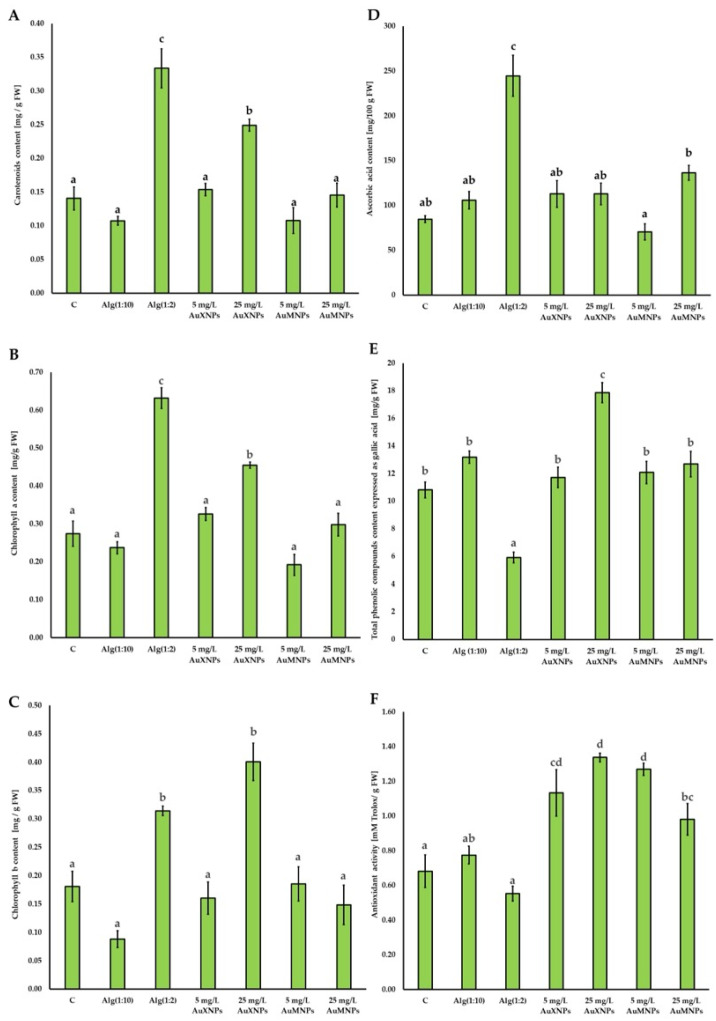
Chemical compounds and antioxidant activity of garden cress seedlings. Individual parameters: (**A**) total carotenoid content, (**B**) chlorophyll *a* content, (**C**) chlorophyll *b* content, (**D**) ascorbic acid content, (**E**) total polyphenolic compounds content, (**F**) level of antioxidant activity. Treatments: C—negative control—distilled water, Alg(1:10)—positive control—sodium alginate without gold nanoparticles, diluted 10-fold, Alg(1:2)—positive control—sodium alginate without gold nanoparticles, diluted 2-fold, 5 mg/L AuXNPs—solution with gold nanoparticles obtained by using xylose as a reducing agent at a concentration of 5 mg/L, 25 mg/L AuXNPs—solution with gold nanoparticles obtained by using xylose as a reducing agent at a concentration of 25 mg/L, 5 mg/L AuMNPs—solution with gold nanoparticles obtained by using maltose as a reducing agent at a concentration of 5 mg/L, 25 mg/L AuMNPs—solution with gold nanoparticles obtained by using maltose as a reducing agent at a concentration of 25 mg/L. Statistically, differences marked with letters (a, b, c, d) differ significantly at *p* ≤ 0.05 according to Fisher’s test. Standard error was used to describe the obtained results.

The treatment with a higher concentration of sodium alginate without AuNPs (Alg(1:2)) significantly increased the carotenoid content of these compounds compared to other treatments with AuNPs and controls: (C, Alg(1:10)) by an average of about 15 mg (Figure 6A). Among the treatments with AuNPs, only nanogold obtained with xylose as a reducing agent at a concentration of 25 mg/L significantly increased the carotenoid content compared to the controls (C, Alg(1:10)) by about 10 mg (Figure 6A).

Sodium alginate without AuNPs (Alg(1:2)) increased the chlorophyll *a* concentration the most compared to other methods, on average about 0.30 mg (Figure 6B). Only treatment with AuNPs obtained by using xylose as a reducing agent at a concentration of 25 mg/L (25 mg/L AuNPs) resulted in an increase in the chlorophyll *a* content compared to other AuNP treatments and the control (C), Alg(1:10), even by an average of 0.15 mg (Figure 6B).

Treatment with an AuNP solution prepared using xylose as a reducing agent at a concentration of 25 mg/L (25 mg/L AuXNPs) and a positive control using sodium alginate without nanogold at a higher concentration (Alg(1:2)) significantly differed in chlorophyll *b* content from all other treatments (Figure 6C). In the case of AuNP treatment, the difference was as much as approximately 0.25 mg, while in the case of the positive control (Alg(1:2)) it was approximately 0.11 mg (Figure 6C). Only the treatment with the AuNP solution prepared using xylose as a reducing agent at a concentration of 25 mg/L (25 mg/L AuXNPs) significantly increased the chlorophyll *b* content compared to the other AuNPs treatments, even by approximately 0.20 mg (Figure 6C).

In the case of ascorbic acid content, only treatment with the positive control, without AuNPs, but with a higher concentration of sodium alginate without AuNPs (Alg(1:2)) significantly increased the content of this compound compared to the other controls (C, Alg(1:10)) and treatments with AuNPs, by approximately 150 mg (Figure 6D). No significant changes were observed between treatments with both AuNP concentrations compared to the negative control (C) and the positive control, with a lower concentration of sodium alginate (Alg(1:10)) (Figure 6D).

In terms of total polyphenolic content, only the positive control with a higher concentration of sodium alginate (Alg(1:2)) significantly reduced the content of these substances compared to all other methods, by up to approximately 10 mg (Figure 6E). Treatment with gold nanoparticles obtained using xylose as a reducing agent at a concentration of 25 mg/L (25 mg/L AuXNPs) significantly increased the total polyphenolic content compared to all other methods, by approximately 8 mg (Figure 6E).

Treatments with all AuNP concentrations significantly increased the level of antioxidant activity of seedlings compared to the negative control (C) and positive controls (Alg(1:10), Alg(1:2)) by approximately 0.8 mM Trolox (Figure 6F). Among the AuNP concentrations tested, nanogold obtained using maltose as a reducing agent at a concentration of 25 mg/L (25 mg/L AuMNPs) caused a significant decrease in antioxidant activity compared to treatment with xylose-derived nanogold at a concentration of 25 mg/L (25 mg/L AuXNPs), and with maltose-derived nanogold at a concentration of 5 mg/L (5 mg/L AuMNPs) by approximately 0.4 mM Trolox (Figure 6F).

## 3. Discussion

Applying the principles of green chemistry makes it possible to reduce the toxicity of metal nanoparticle synthesis by using biodegradable matrices and low-toxicity reducing agents. Previous studies have demonstrated that polysaccharides such as starch, sodium alginate, or other biopolymer systems can act as both stabilizing matrices and as media for the formation of metallic nanoparticles [18,21,22,39,44]. In the present study, sodium alginate served as an effective matrix for the synthesis of AuNPs reduced with either xylose or maltose, while the choice of reducing sugar clearly influenced the nanoparticle morphology, size distribution, and colloidal behavior.

TEM analysis showed that both formulations contained predominantly quasi-spherical nanoparticles; however, the xylose-based system exhibited a narrower size distribution and a lower proportion of particles larger than 30 nm than the maltose-based system. This suggests that xylose promoted a more controlled reduction and nucleation process, resulting in a more homogeneous nanoparticle population. In contrast, the broader distribution observed for the maltose-derived sample may reflect a less synchronized nucleation stage and a wider time window for nanoparticle growth. This interpretation is consistent with previous reports showing that the chemical identity of the carbohydrate reductant can substantially affect the kinetics of AuNP formation, particle morphology, and polydispersity in polysaccharide-based systems [18,39,45,46,47,48,49].

The EDS results additionally strengthened the structural interpretation of the TEM images by confirming that the electron-dense domains corresponded to Au-containing nanostructures rather than artifacts of the polymer matrix. Although EDS was presented here as a representative analysis, the obtained spectra support the conclusion that gold was successfully incorporated into the analyzed alginate-based nanocomposite [33].

A clear difference was also observed between the particle dimensions obtained by TEM and the hydrodynamic diameters measured by DLS. This discrepancy is expected because TEM visualizes only the electron-dense metallic core in the dry state, whereas DLS measures the hydrodynamic diameter of the particle together with the surrounding solvation layer and polymer-associated shell. In the present system, the alginate matrix and residual sugar-derived surface components most likely contributed to the formation of an extended hydrated shell, which increased the apparent particle size in solution. This effect was particularly pronounced for the maltose-reduced formulation, in which the DLS diameter reached 82 nm, compared with 58 nm for the xylose-reduced sample [33,49].

The zeta potential data provide an additional explanation for these differences. The more negative zeta potential of AlgAuXNPs (−38.5 mV) indicates stronger electrostatic repulsion between particles, and consequently, better colloidal stabilization. In contrast, the less negative value recorded for AlgAuMNPs (−32.5 mV) suggests reduced electrostatic stabilization and a greater tendency toward the formation of loose aggregates or more compact polymer-associated structures in solution. This interpretation is consistent with both the broader TEM size distribution and the larger hydrodynamic diameter observed for the maltose-based system [33].

From a functional point of view, the narrower particle size distribution, lower hydrodynamic diameter, and more negative zeta potential observed for the xylose-based formulation suggest a more stable and structurally uniform colloidal system. Such differences may be important for subsequent biological interactions, because nanoparticle size, dispersity, and surface charge are among the key parameters governing interfacial behavior and bioactivity in plant exposure systems [33,50]. The physicochemical differences observed here therefore provide a plausible basis for the distinct biochemical responses recorded later in garden cress seedlings.

The applied AuNP formulations also influenced selected morphological traits of garden cress seedlings, particularly root length. In the present study, shoot length was not significantly affected relative to the negative control, whereas root growth was reduced after exposure to several formulations, especially those containing AuNPs synthesized with maltose and the higher alginate control. This suggests that root development was more sensitive than shoot growth under the applied experimental conditions. Similar variability in plant growth responses to AuNP exposure has been reported in the literature and appears to depend on plant species, nanoparticle concentration, exposure route, and experimental system. The inhibitory effect observed for root growth may also partly reflect the physical properties of the alginate matrix, particularly at the higher concentration, which may have limited seedling development on filter paper.

AuNP exposure may also influence selected growth-related parameters of seedlings, including shoot and root length. Tomaszewska-Sowa et al. [28] reported that AuNP treatment reduced the shoot length in rapeseed seedlings, whereas Ferrari et al. [51] observed the stimulation of root growth in *Arabidopsis thaliana*, and Sahil et al. [52] found enhanced shoot and root growth in pearl millet after AuNP exposure. In the present study, none of the tested formulations significantly affected the shoot length relative to the negative control, whereas root growth was reduced by most treatments, indicating that root development was more sensitive than shoot growth under the applied experimental conditions. The reduction observed for the higher alginate control suggests that, apart from nanoparticle-related effects, the physical properties of the alginate matrix may also have contributed to the limitation of seedling growth [22].

The concentration of individual photosynthetic pigments may depend on the method of treatment of plants with nanoparticles [21,53]. Peshkova et al. [54] reported that the content of photosynthetic pigments varied depending on the concentration of AuNPs applied by watering potted *Petroselinum crispum* plants. After the application of low concentrations of AuNPs (1 and 5 mg/L), an increase in the content of total carotenoids and chlorophylls (by about 12.7%) was observed; the application of high concentrations of AuNPs (above 10 mg/L) resulted in a decrease in the content of these pigments [54]. Manaf et al. [55] documented that the use of AuNPs at concentrations of 20 and 30 mg/L reduced the total chlorophyll content in wheat sprayed with nanoparticles. Tomaszewska-Sowa et al. [28] found that in *Brassica napus* L. seedlings grown in vitro, the total chlorophyll content decreased after the application of AuNPs (100 mg/L) during the first seven days of plant cultivation by approximately 0.31 mg [28]. Treatment with two analyzed concentrations of nanogold (50 and 100 mg/L) did not cause a significant reduction in the carotenoid content in *Brassica napus* L. seedlings [28]. Interestingly, in the case of *Mentha spicata* L., spraying AuNPs on the leaves stimulated an increase in chlorophyll content at concentrations of 1 and 5 mg/L [29]. Treatment of *Spinacia oleracea* L. seeds with gold nanoparticles at a concentration of 150 μM resulted in an increase in the content of chlorophyll *a* by about 44.18%, chlorophyll *b* by about 82.76%, and total carotenoids by 64.95% compared to the control treatment in spinach [30]. Treatment of wheat seeds *Triticum aestivum* L. [31] by soaking them in solutions with AuNPs and germinating them in distilled water for 10 days resulted in obtaining seedlings characterized by an increase in the content of chlorophyll *a* by about 8% and chlorophyll *b* by about 17%. The presence of AuNPs did not affect the total carotenoid content of *Triticum aestivum* L. [31]. Our study did not reveal a significant reduction in the content of photosynthetic pigments, carotenoids, chlorophyll *a*, and chlorophyll *b*, after exposure to the two tested concentrations of AuNPs in garden cress. However, we demonstrated that the presence of AuNPs at a concentration of 25 mg/L significantly increased the content of carotenoids and chlorophyll *a* and *b*. The differences in our results are likely due to different plants used or different forms of nanoparticle application compared to the results of the researchers described in this discussion.

Sodium alginate treatment may also affect the content of photosynthetic pigments. In red cabbage, treatment with sodium alginate solutions resulted in the accumulation of higher carotenoid content. Chlorophyll accumulation was also observed after sodium alginate treatment [22]. Our results also indicate the stimulation of all photosynthetic pigments by treatment with a sodium alginate solution without AuNPs. This may be due to the temporary decomposition of sodium alginate into simpler sugar compounds, which are an energy source for garden cress seedlings.

The use of AuNPs may potentially influence the variability of the concentration of individual bioactive compounds associated with antioxidant processes in plant tissue. Jurkow et al. [56] documented that foliar application of AuNPs at concentrations of 10 and 20 mg/L did not significantly affect the concentration of L-ascorbic acid in oakleaf lettuce. The presence of AuNPs also did not affect the content of polyphenolic compounds or antioxidant activity. Joshi et al. [57] found that the use of different concentrations of AuNPs (40 and 60 µM) increased the total polyphenol content in *Nardostachys jatamansi* in vitro. However, Jadczak et al. [58] found that the content of polyphenolic compounds in lavender (*Lavandula angustifolia* Mill.) propagated in vitro varied depending on the concentration of gold nanoparticles used. At low concentrations (2 or 5 mg/L), an increase in the total polyphenol content was observed, whereas at higher concentrations (20 or 50 mg/L), the concentration of polyphenolic compounds decreased significantly [58].

All tested AuNP concentrations significantly increased the antioxidant activity of the seedlings. This response may indicate the activation of antioxidant and photoprotective defense pathways, however, it should not be interpreted as direct evidence of oxidative damage, because direct markers of oxidative damage and ROS metabolism were not assessed in the present study. In plant tissues, exposure to nanoparticles may transiently disturb the balance between reactive oxygen species production and scavenging, leading to a transient increase in superoxide radicals and hydrogen peroxide. In turn, plants activate antioxidant defense mechanisms to restore redox equilibrium. These responses involve both enzymatic antioxidants and non-enzymatic metabolites, including ascorbic acid, polyphenolic compounds, carotenoids, and other pigments associated with photoprotection and stress acclimation [53,55,56,57,58]. Therefore, the changes observed in antioxidant activity and selected bioactive compounds in the present study are consistent with the activation of defense-related pathways associated with ROS clearance. At the same time, because direct markers of oxidative damage and ROS metabolism were not determined, these results should be interpreted as evidence of stress-related biochemical adjustment rather than direct proof of oxidative injury [58]. The observed differences compared to the literature data [28,29,30,31,55] may likely result from the tested AuNP concentrations, the form of their application, and species-specific plant response. In addition, sodium alginate itself contributed to some of the observed biochemical changes, as alginate and alginate-derived oligosaccharides are recognized as biologically active compounds [59]. Rutkowski et al. [22] documented an increase in ascorbic acid content in red cabbage after treatment with solutions containing sodium alginate solutions, however, the presence of this polysaccharide did not significantly affect the content of polyphenolic compounds or antioxidant activity. A similar pattern was observed in our study, where the higher concentration of sodium alginate increased the content of ascorbic acid, decreased the polyphenolic compounds, and did not modify the antioxidant activity. These findings suggest that the alginate matrix may influence selected biochemical responses of seedlings, which is consistent with the reports of Vera et al. [59] and Shukla et al. [60].

In our opinion, the observed differences among formulations synthesized with maltose and xylose suggest differences in their surface-associated biological activity, as reported by Daniel and Astruc [50]. In the present study, the control design did not include alginate systems supplemented separately with xylose or maltose in the absence of nanoparticle formation; therefore, the contribution of residual sugar-derived components to the observed plant responses cannot be entirely excluded. Reducing sugars used during nanoparticle synthesis may act not only as reducing agents, but also as surface-stabilizing molecules that influence the NP surface chemical properties [61] and consequently their interactions with plant tissues [41], particularly the induction of oxidative stress and related metabolic reactions. Chlorophylls and carotenoids are highly sensitive to cellular redox imbalances, while ascorbic acid and polyphenolic compounds constitute important non-enzymatic components of the antioxidant defense system [57,58]. Therefore, the differential response of these metabolites likely reflects differences in the intensity of stress induced by AuNP formulations, suggesting xylose as the factor responsible for the stronger response in seedlings.

## 4. Materials and Methods

### 4.1. Reagents Used During Synthesis

Sodium alginate (mass ≈ 1.565 × 105 Da); chloroauric acid (99.99%); D(-)-maltose (≥99%); D(-)-xylose (≥99%); glycerol (99.5%). All reagents were purchased from Sigma-Aldrich (Poznań, Poland).

### 4.2. Synthesis of Alginate Gels with Gold Nanoparticles

The preparation of gold nanoparticles was performed in a manner similar to that described in Janik et al. [18] using a single reagent composition, as shown in Table 2.

Alginate gel (2%) was gelatinized by dissolving 2 g of sodium alginate (Table 2) in 98 g of distilled water (Table 2) until a homogeneous suspension was obtained at 80 °C for 24 h, adding glycerol as a plasticizer in a weight ratio of 1:2 to the alginate mass (Table 2). The obtained gel was divided into three equal parts. To obtain the same alginate concentration, 4.6 g of distilled water was added to the first sample (AlgC) (Table 2). To the second sample (AlgAuXNPs), 2.6 g of chloroauric acid solution (HAuCl_4_ 0.01 M) (Table 2) and 2 g of reducing sugar solution (xylose, 4%) (Table 2) were added. To the third sample (AlgAuMNPs), 2.6 g of chloroauric acid solution (HAuCl_4_ 0.01 M) (Table 2) and 2 g of reducing sugar solution (maltose, 4%) (Table 2) were added. All samples were further stirred on a magnetic stirrer at 80 °C until the suspension turned purple, characteristic of gold nanoparticles. This resulted in the production of alginate gels with gold nanoparticles at an initial concentration of 50 mg/L (Table 2) and a gel without nanometals, which were then analyzed for their physicochemical properties.

### 4.3. Physicochemical Evaluation of Alginate Gels with Gold Nanoparticles

The morphology and elemental composition of the gold nanoparticles were evaluated by scanning electron microscopy (SEM) coupled with energy-dispersive X-ray spectroscopy (EDS), using a JEOL JSM-7500F microscope (JEOL, Tokyo, Japan) connected to an AZtecLiveLite Xplore 30 system (Oxford Instruments, Abingdon, UK). The instrument was equipped with a transmission electron detector (TED) and a retractable backscattered-electron detector (RBEI). The hydrodynamic size and zeta potential (ζ) of the gold nanoparticles were determined by dynamic light scattering (DLS) using a Zetasizer Nano ZS instrument (Malvern Instruments Ltd., Malvern, UK).

Alginate gels were diluted tenfold with distilled water prior to UV–Vis measurements. UV–Vis absorption spectra were recorded in the 200–700 nm range using a Shimadzu 2101 scanning spectrophotometer (Shimadzu, Kyoto, Japan). Measurements were performed in a quartz cuvette, with distilled water used as the reference.

For attenuated total reflectance-Fourier transform infrared (ATR-FTIR) analysis, 25 g of each gel sample was poured into Petri dishes and dried at 37 °C to obtain thin films. The spectra of the control sample and the films containing gold nanoparticles were recorded over the range 4000–700 cm^−1^ at a resolution of 4 cm^−1^. ATR-FTIR measurements were carried out using a MATTSON 3000 FTIR spectrophotometer (Madison, WI, USA) equipped with a 30SPEC 30° reflective shield and a MIRacle ATR accessory from PIKE Technologies Inc. (Madison, WI, USA).

### 4.4. Experimental Design

The initial alginate gels with AuNPs were diluted 10-fold and 2-fold to obtain two concentrations of gold nanoparticles: 5 and 25 mg/L. The gel without gold nanoparticles was also diluted in the same volumetric manner: Alg(1:10) and Alg(1:2). These two alginate-only controls corresponded directly to the same dilution ratios as those used for the AuNP-containing formulations, enabling the effects of the nanoparticle-containing systems to be interpreted against matched alginate backgrounds. Then, 10 mL of the tested solutions were added to plates filled with filter paper. Plates with distilled water were designated as a negative control (C). A total of 1 g of garden cress seeds was sown in each plate. Each treatment was performed in triplicate (n = 3). The seeds were incubated for 5 days at room temperature, with access to light. After this time, whole garden cress seedlings were harvested. The mean shoot and root lengths were measured using a Yato caliper, and the values of individual biochemical parameters were then determined, including photosynthetic pigment content (carotenoids, chlorophyll *a*, and chlorophyll *b*), ascorbic acid content, polyphenolic compound content, and antioxidant activity.

In the experiment with plant seedlings, the following seed treatment methods were tested:

- C—negative control—distilled water;
- Alg(1:10)—positive control- sodium alginate without gold nanoparticles, diluted 10-fold;
- Alg(1:2)—positive control—sodium alginate without gold nanoparticles, diluted 2-fold;
- 5 mg/L AuXNPs—solution with gold nanoparticles obtained by using xylose as a reducing agent at a concentration of 5 mg/L;
- 25 mg/L AuXNPs—solution with gold nanoparticles obtained by using xylose as a reducing agent at a concentration of 25 mg/L;
- 5 mg/L AuMNPs—solution with gold nanoparticles obtained by using maltose as a reducing agent at a concentration of 5 mg/L;
- 25 mg/L AuMNPs—solution with gold nanoparticles obtained by using maltose as a reducing agent at a concentration of 25 mg/L.

### 4.5. Determination of the Biochemical Characteristics of Garden Cress

The content of total carotenoids, chlorophyll *a*, and chlorophyll *b* was determined using the method of Lichtenthaler and Wellburn [62]. Fresh, whole seedlings (0.1 g) were thoroughly homogenized with 25 mL of 80% (*v*/*v*) acetone with the addition of magnesium carbonate (3 mg). The samples were then tightly closed and left to incubate in the dark for 30 min at room temperature. The resulting suspension was filtered through filter paper (POCH S.A., Gliwice, Poland). Absorbance measurements were performed at wavelengths of 646, 663, and 470 nm using a Helios Beta UV–Vis spectrophotometer (Waltham, MA, USA) to determine the concentrations of individual pigments.

The iodometric technique of Ikewuchi and Ikewuchi [63] was used to determine the L-ascorbic acid content in plant material. A sample of fresh garden cress seedlings (1.25 g) was homogenized with 10 mL of 1% oxalic acid and incubated for 30 min at room temperature in the dark. After this time, 5 mL of filtered extract containing 1 mL/L of 1% starch was titrated with potassium iodide solution. As long as ascorbic acid was present in the solution, it reacted regularly with iodine, and the starch was colorless. The iodine remaining after oxidation of the ascorbic acid contained in the sample formed a blue complex with starch, indicating the end of the titration.

Polyphenolic compounds were determined using the Folin–Ciocalteau method described by Djeridane et al. [64]. Fresh garden cress seedlings (2 g) were homogenized with 10 mL of 80% methanol and then centrifuged (3.492 g for 10 min at 18 °C) for 5 min at room temperature. Plant extracts (0.1 mL) were mixed with 2 mL of 2% sodium carbonate. After 2 min, Folin–Ciocalteau reagent (0.1 mL) mixed with deionized water (1:1 *v*/*v*) was added to the tubes. The mixture was incubated at room temperature (20 °C) in the dark for 45 min. Absorbance was measured at 750 nm using a Helios Beta UV–Vis spectrophotometer (Waltham, MA, USA).

The antioxidant activity of garden cress seedlings was determined by the spectrophotometric method using stable free radicals 2,2-diphenyl-1-picrylhydrazyl (DPPH^•^) at a wavelength of 517 nm [65]. A total of 2.95 mL of 0.1 mM DPPH solution (Sigma–Aldrich) in 96% ethanol was mixed with 0.05 mL of methanolic plant extract. The extracts were prepared in the same manner as for the determination of the polyphenol content. DPPH absorbance was measured after 5 min using a Double Beam U-2900 spectrophotometer (Hitachi High-Technologies Corporation, Japan).

### 4.6. Statistical Analysis

Statistical significance of differences was calculated using one-way analysis of variance, and means were separated into homogeneous groups using Fisher’s least significant difference (LSD) test at *p* < 0.05 using Statistica software, version 13.3 (TIBCO Software Inc., Palo Alto, CA, USA). Data presented in the accompanying bar graphs represent the means and standard errors. The absence of letters in the figure indicates no statistically significant differences between the objects.

## 5. Conclusions

This study demonstrated an eco-friendly method for the synthesis of gold nanoparticles (AuNPs) in a sodium alginate matrix using maltose and xylose as reducing agents. Physicochemical characterization, including TEM, EDS, DLS, zeta potential, and UV–Vis analysis, showed that the choice of reducing sugar influenced the size, dispersity, colloidal behavior, and optical properties of the resulting nanoparticles. In garden cress (*Lepidium sativum* L.) seedlings, the tested AuNP formulations did not cause any clear inhibitory effects in the analyzed biochemical endpoints under the tested conditions, but they did modify selected biochemical parameters, and these effects depended on both the nanoparticle concentration and the reducing sugar used during synthesis. In particular, AuNPs synthesized with xylose at a concentration of 25 mg/L significantly increased the content of photosynthetic pigments (chlorophyll *a*, chlorophyll *b*, and total carotenoids) and polyphenolic compounds. All tested AuNP formulations increased the antioxidant activity of garden cress seedlings, which is consistent with the activation of stress-related defense responses. Overall, the results indicate that alginate-based AuNP formulations can modify selected biochemical parameters in garden cress seedlings, with the observed effects depending on the formulation characteristics. However, since oxidative damage markers and nanoparticle uptake and distribution were not analyzed, the mechanistic basis of these responses requires future investigation. Nevertheless, the tested AuNP formulations may be considered as promising plant-targeted nanomaterials capable of modifying the biochemical status of seedlings, with potential relevance for the development of new agricultural materials.

## Figures and Tables

**Table 1:** Size distribution parameters of alginate-based gold nanoparticles synthesized using xylose and maltose as reducing agents.

Sample	*n*	Mean Diameter ± SD [nm]	Median [nm]	Range [nm]	CV * [%]	Particles > 30 nm [%]
AlgAuXNPs (xylose)	28	23.1 ± 7.2	23.9	8.1–34.4	31.2	21.4
AlgAuMNPs (maltose)	27	25.8 ± 10.4	28.3	7.2–40.3	40.2	40.7

* CV, coefficient of variation.

**Table 2:** Content of reagents necessary for the synthesis of gold nanoparticles in sodium alginate. AlgC—control gel without gold nanoparticles, AlgAuXNPs—gel containing gold nanoparticles obtained by using xylose as a reducing agent, AlgAuMNPs—gel containing gold nanoparticles obtained by using maltose as a reducing agent.

Samples	Water [g]	Sodium Alginate [g]	HAuCl_4_ (0.01 M) [g]	Glycerol [g]	Xylose Solution 4%, [g]	Maltose Solution 4%, [g]	Total Mass of Gel [g]	AuNP Concentration [mg/L]
AlgC	102.60	2.00	0.00	1.00	0.00	0.00	105.60	0.00
AlgAuXNPs	98.00	2.00	2.60	1.00	2.00	0.00	105.60	50.00
AlgAuMNPs	98.00	2.00	2.60	1.00	0.00	2.00	105.60	50.00

## Data Availability

The original contributions presented in this study are included in the article. Further inquiries can be directed to the corresponding author.
